# Methicillin resistant and susceptible *Staphylococcus aureus*: Appraising therapeutic approaches in the Northwest of Iran

**Published:** 2013-03

**Authors:** Alka Hasani, Vajihe Sheikhalizadeh, Akbar Hasani, Behrouz Naghili, Vahide Valizadeh, Ali Reza Nikoonijad

**Affiliations:** 1 Research Center of Infectious Diseases and Tropical Medicine, Departments of Medical Microbiology; 2 Departments of Medical Microbiology; 3 Biochemistry and Laboratory Sciences; 4 Infectious Diseases, Faculty of Medicine, Tabriz University of Medical Sciences, Tabriz, Iran; 5 Department of Pharmaceutical Biotechnology, Pasteur Institute of Iran; 6 Department of Infectious Diseases, Urumieh University of Medical Sciences, Urumieh, Iran

**Keywords:** *Staphylococcus aureus*, increased antibiotic resistance, MIC

## Abstract

**Background and Objectives:**

*Staphylococcus aureus* is a versatile organism causing mild to life threatening infections. The major threat of this organism is its multidrug resistance. The present study was carried out to investigate *in - vitro* activity of conventional antibiotics routinely prescribed for methicillin resistant *S. aureus* (MRSA) and methicillin sensitive *S. aureus* (MSSA) infections in the Northwest of Iran and other alternating therapeutic agents which are recommended for Gram positive organisms.

**Materials and Methods:**

Clinical isolates of *S. aureus* were subjected to multiplex PCR for simultaneous speciation and detection of methicillin resistance. Antibacterial susceptibility pattern was determined using disk diffusion. The Minimum Inhibitory Concentrations (MICs) were determined using E-test strips.

**Results:**

The results revealed presence of *nuc* gene in all *S. aureus* isolates detected phenotypically earlier whereas, *mec*A gene was observed in 54% of strains. On disk diffusion and MIC determination assay, all MRSA and MSSA strains were susceptible to mupirocin (except one MRSA strain), linezolid and teicoplanin. Six vancomycin intermediate *S. aureus* strains were detected (VISA) with MIC= 4µg/mL, 5 of them being MRSA. In disk diffusion assay, 17.3% and 3.7% of isolates showed resistance to rifampin and fusidic acid, respectively. However, MIC_50_ and MIC_90_ tests shows promising *in – vitro* impact.

**Conclusion:**

In *–* vitro mupirocin was found as an effective prophylactic ointment for nasal *S. aureus* eradication. Our data emphasize the performance of surveillance exercises to outline the existing antibiotics prescription policies and to slow down the emergence of multidrug resistant strains.

## INTRODUCTION

*Staphylococcus aureus* is the foremost nosocomial pathogen facing humans today. Since the first isolation of methicillin resistance in *S. aureus* in 1961, the realm of concern is about its expanded prevalence, along with its efficiency at developing resistance to other antimicrobial agents. Until recently, vancomycin was considered the antibiotic of choice either solely or in combination, however, emergence of vancomycin intermediate-resistant *S. aureus* in Japan was followed by awareness of similar strains worldwide (1).

In the last few years, published reports have introduced antibacterial agents such as linezolid, fusidic acid, rifampin, and teicoplanin to treat various infections caused by MRSA (2). Nevertheless, for an effective approach, it is mandatory that antimicrobial agents must have activity against antibiotic-resistant *S. aureus*, along with low potential for resistance development.

Though vancomycin is a drug of choice for MRSA infections in hospitalized patients, its reduced susceptibility and poor tissue penetration make its therapeutic efficacy a concern. Linezolid has been compared with vancomycin and has been found equivalent in terms of tolerance and superior to vancomycin in treatment of complicated skin and soft tissue infections due to suspected or confirmed MRSA (3, 4). Linezolid resistance is uncommon.The year 2010 witnessed its first clinical outbreak with Linezolid Resistant *S. aureus* (LRSA). Nosocomial transmission and extensive linezolid usage were the factors associated with the outbreak. Usage and infection control measures were the suggestions to overcome such situation (5).

Fusidic acid has been found to possess equal or greater potency against staphylococci compared with vancomycin or daptomycin (6). The drug has been potentially useful as a topical agent for skin infections and proven effective for difficult – to – treat MRSA infections. Fusidic acid-resistant *S. aureus* has been reported in many countries, with the prevalence ranging from 0.3 to 52.5%, however, in Iran, MRSA and MSSA strains have been reported to be susceptible (7).

Rifampin, has also been an attractive broad spectrum antimicrobial choice for treating *S. aureus* infections, however, the drug is always proposed adjunctively (8). Its usage as oral therapy has been suggested for eradication of *S. aureus* carriage. Rifampin resistant *S. aureus* has been reported in Iran, with the prevalence ranging from 8-17% (9).

Vancomycin and mupirocin have been employed against *Staphylococcus aureus* in our region, however, other antibacterial agents have not been in conventional usage.

The aim of the present study was to determine comparative activities of oxacillin, vancomycin, mupirocin, rifampin, fusidic acid, linezolid and teicoplanin against clinical isolates of methicillin resistant and susceptible *Staphylococcus aureus* strains by disk diffusion and E-test in our region.

## MATERIAL AND METHODS

### Isolation and Identification of *S. aureus*

In an analytic–descriptive cross sectional study carried out at University Teaching Hospital, serving as the referral center for patients from North West region of Iran, a total of 1,945 clinical specimens, including blood, urine, postoperative wound, synovial fluid, sputum and anterior nares were processed for the isolation and identification of *Staphylococcus aureus* according to phenotypic methods such as Gram's staining, yellow or white colonies on blood agar (yellow colonies on mannitol salt agar for nasal swabs), catalase, slide and tube coagulase and DNase tests (10). Duplicate isolates from the same patient were not included. All isolates were immediately stored at -70°C until required.

### PCR for speciation and methicillin resistance

All isolates were confirmed as *S. aureus* by screening for the nuclease – encoding gene (*nuc*) and for methicillin resistant by *mecA* by using a multiplex PCR (11, 12). Primers were synthesized by Eurofin, Germany. Strains were considered as MRSA or MSSA based on the presence or absence of *mec*A gene respectively.

Briefly, DNA was extracted using SDS-Proteinase K with CTAB method as prescribed by Sambrook and Russell (13). Multiplex PCR (mPCR) was performed in 25 µl PCR reaction mixture containing; 1X PCR buffer, 1.2 mM MgCl_2_, 0.2 deoxynucleoside triphosphates sets, 0.5 mM each oligonucleotide primer and 2.5 U of *Taq* polymerase. The PCR reaction was as follows: an initial denaturation at 95°C for 2 min, with 30 cycles of denaturation at 94°C for 30s, annealing at 58°C for 30s, extension at 72°C for 45s and final extension at 72°C for 5 min. The *mec*A specific PCR product was 154 bp long and the presence of *nuc* gene was observed with an expected size of 270 bp. *S. aureus* ATCC 29213 was used as control strain.

### Antimicrobial testing Qualitative evaluation

Susceptibility testing for MRSA and MSSA was conducted on Mueller-Hinton agar by disk diffusion technique according to the guidelines of Clinical Laboratory Standards Institute (CLSI) (14) with a panel of following antibiotics: oxacillin (1 µg), vancomycin (30µg), teicoplanin (30 µg), linezolid (30 µg), rifampin (30 µg), mupirocin (5 µg) and fusidic acid (10 µg), all purchased from MAST (UK). As there are no available CLSI interpretive criteria for fusidic acid and mupirocin for *S. aureus*, susceptible phenotype defined as a zone diameter of 22 mm (15) and ≥14 mm (16) was used respectively. *S. aureus* ATCC 25923 and ATCC 43300 strains were used as controls for the antibiotic susceptibility determination.

### Quantitative evaluation

The MICs were determined on Mueller-Hinton agar plates for oxacillin, vancomycin, teicoplanin, linezolid, rifampin, mupirocin and fusidic acid by standard E-test method according to the manufacturer's recommendations (bio Mérieux, Inc). The breakpoints for resistance were those defined by the CLSI (14), except for mupirocin and fusidic acid for which breakpoints from the study of Finlay *et al*. (15) (MIC ≤4µg/ ml as susceptible) and European Society of Microbiology (16) which suggests MIC ≤ 1 as susceptible and MIC > 1 as resistant,were used.

## RESULTS

Among the total 1,945 clinical specimens processed, 150 *S. aureus* were isolated after identification on the basis of phenotypic tests, all strains scored positive for the *nuc* gene, while *mecA* gene was revealed in 81 (54%) isolates (considered as MRSA), and the remaining 69 (46%) isolates were identified methicillin sensitive (MSSA). The source of these isolates was as follows: surgical and internal wards (n = 51), burn patients (n = 36), infectious ward (n = 25), skin and hemodialysis (n = 7 each) and the remaining isolates (n = 24; 14.6%) were obtained from various ICU's. Concerning the origin of MRSA isolates, majority of strains [n = 46 (56.7%)] were isolated from wounds, followed by bloodstream [17 (20.9%)], endotracheal tube [8 (9.8%)], nasopharynx (n = 4;9%), synovial fluid (n = 3; 7%), and the remaining were obtained from specimens like intravenous catheter, and other body fluids.

Similarly, MSSA isolates were obtained from postoperative wound [n = 29; (42%)], bloodstream [n = 21; (30.4%)], endotracheal tube (n = 9; 13.04%), body fluids (n = 3; 4.34%) and remaining from other clinical specimens like nasopharynx, synovial fluid, urine and intravenous catheter.

On disk diffusion assay, *me*cA-positive MRSA strains revealed 88.8%, 17.3% and 3.7% as being resistant to oxacillin, rifampin and fusidic acid, respectively. Only one isolate (1.23%) was observed resistant to mupirocin. All MRSA strains were found sensitive to teicoplanin. Among MSSA strains, all isolates were uniformly found sensitive to fusidic acid and mupirocin, while few of them showed non susceptibilty to rifampin (2.9%). Surprisingly, 8.7% MSSA (*mec*A gene not detected) were found resistant to oxacillin on disk diffusion. All isolates of *S. aureus* including MRSA and MSSA, were found sensitive to linezolid by disk diffusion (Table 1).

**Table 1:** *In- vitro* activities of tested antimicrobial agents against 81 mecA-positive MRSA and 69 mec- negative MSSA strains.

Antibiotics	Disk Diffusion^a^						E- test^a^					

	MRSA (n= 81)			MSSA(n= 69)			MRSA(n= 81)			MSSA(n= 69)		

	R	I	S	R	I	S	R	I	S	R	I	S
**Oxacillin**	88.8	4.9	6.2	8.7	0	91.3	96.2	0	3.7	b 4.3	0	95.6
**Vancomycin**	0	55.5	44.4	0	4.3	95.6	0	35.8	64.2	0	27.5	72.5
**Rifampin**	17.3	4.9	77.7	2.9	0	97.1	17.3	3.7	79.0	2.9	2.9	95.2
**Fusidic acid**	3.7	0	96.2	1.2	0	98.5	4.93	0	95.0	4.34	0	95.6
**Mupirocin**	1.23	0	98.7	0	0	100	1.23	0	98.7	0	0	100
**Teicoplanin**	0	0	100	0	1.4	98.5	0	0	100	0	0	100
**Linezolid**	0	0	100	0	0	100	0	0	100	0	0	100

a R: resistant; I: intermediate; S: sensitive

b All oxacillin resistant isolates had MICs≥ 256 mg/l.

**Fig. 1:**
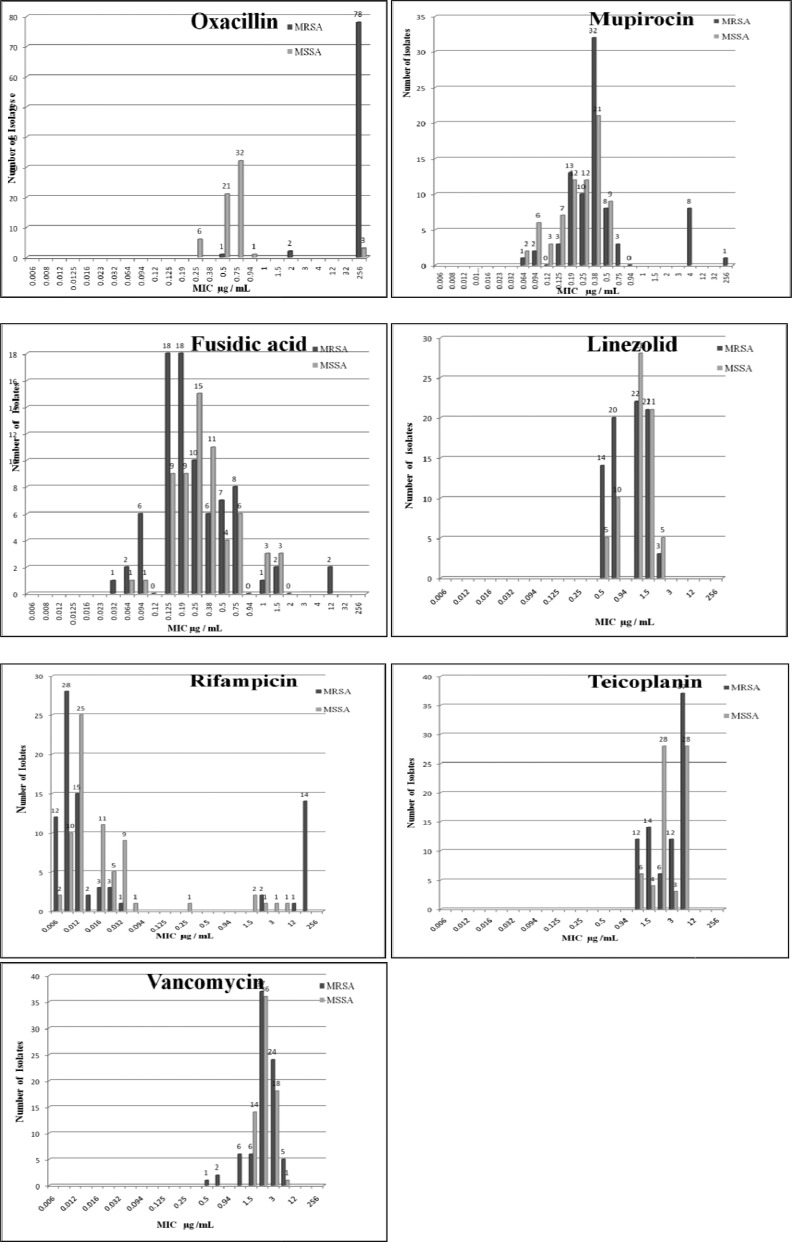
Distribution of MIC values of MRSA and MSSA isolates for various antibiotics.

Interestingly, 55.5% MRSA and 4.3% MSSA strains produced a zone diameter equal to 14mm for vancomycin on disk diffusion assay, which should be reported as intermediate, however, when MIC assay was performed by E-test, MIC ranged from 0.5- 4 µg/mL and taking into consideration the interpretive criteria of vancomycin which has changed since 2006 when MIC breakpoints for each category reduced one-fold, 48 (32%) strains were observed as VISA. Of these, 6 isolates had MIC = 4 µg/mL (8.3%) (all of them being MRSA), while 42 isolates (24 of them being MRSA and 18 MSSA) were observed revealing MIC = 3µg/mL, which is one log higher than the susceptible level (MIC ≤ 2µg/mL) Though our study did not find a very high level vancomycin resistance (MIC_50_ =1; MIC_90_=1.8), however, this upward MIC shift from level of susceptible towards intermediate level is *in vitro* concern. Presence of six VISA strains or a slightly upward shift in MIC level has not yet impacted significantly in vivo but shows the occurrence of VISA in vitro in our environment. Among the strains which showed MIC = 2 µg/mL (n = 73), 36 of them were MRSA. The MICs of different antimicrobial agents for MRSA and MSSA strains are presented in Tables 1 and 2 respectively and Fig. 1.

Among MRSA strains, all were found highly resistant (MIC ≥256 mg/L) to oxacillin (MIC_50_=128 µg/mL; MIC_90_=256 µg/mL). Mupirocin resistance was not seen in any of the MRSA isolates except one isolate showing MIC = 128 mg/L. This mupirocin resistant strain was highly resistant to oxacillin (≥256 mg/L), rifampicin and fusidic acid (each with MIC = 12 mg/L). In comparison, MSSA isolates had MIC_50_=0.5 µg/mL and MIC_90_=0.9 µg/mL)

MICs for fusidic acid for 7 isolates was over 1 mg/L (two MRSA isolates with MIC= 12 mg/L, two MRSA and 3 MSSA isolates with MIC = 1.5 mg/L) (Fig. 1) thus, though were considered as resistant, however, MIC_50_ and MIC_90_ did not reveal concern (MIC_50_= 0.9 mg/L; MIC_90_=0.5 mg/L). The characterization of fusidic acid-resistant detected by the E-test is presented in 3.

Twenty one (14%) isolates (17 being MRSA while, 4 MSSA) were observed to have MIC >1 for rifampin (Fig. 1), thus were considered resistant, however, overall the antibiotic was shown a potential impact with MIC_50_ and MIC_90_ of MRSA isolates being 0.006 mg/Land MIC_90_=0.02 mg/Lrespectively. Teicoplanin and linezolid provided promising activity for all MRSA and MSSA isolates (Fig. 1).

## DISCUSSION

*Staphylococcus aureus* is particularly efficient at developing resistance to antimicrobial agents and introduction of new class of antimicrobial agents has been followed by the emergence of resistant forms of this pathogen (1). In view of expanded use of antibiotics, there is always a need to survey antibiotic pattern to comprehend emerging trends.Microbiological laboratories play an important role in characterization of the pathogens, detection and confirmation of any emergence of antibiotic resistance.

In the present study, prevalence of methicillin resistant *S. aureus* was 54% (with *mec* A gene detected), which is in concordance with the studies conducted by Yadegar *et al*. (17) in Tehran, Iran and Stenstorm *et al*. (18) in Canada, but was higher compared to Shittu *et al*. (19) survey from South Africa. Since the specimens collected in our study were isolated from patients admitted to high risk wards of a University affiliated referral hospital serving for North West region of Iran, such a high prevalence requires attention and should not be ignored. MRSA does not appear to be more virulent, however it possess more risk in terms of resistance to other antibiotics.

MIC determinations of oxacillin in current study showed 98.3% of MRSA isolates were highly resistant to oxacillin (MICs ≥256 mg/l) while all harbored *mecA* gene. Surprisingly, three (4.3%) MSSA strains (*mecA* negative) were observed highly resistant to oxacillin with MIC ≥256 mg/l. The absence of *mecA* gene in these strains indicates an alternative mechanism of oxacillin resistance such as the β- lactamase hyperproduction (20) or production of normal PBP with altered binding capacity (21). On the other hand, one MSSA isolate which had MIC value equal to 0.75 mg/l, later was found to possess *mecA* gene. The occurrence of this variant could be explained by the presence of complete regulator genes (*mecI* and/or *mecRI*), as described previously (22). The emergence of vancomycin intermediate-resistant *S. aureus* is a great concern and has been proposed to pose a serious challenge to the clinicians in finding an alternative treatment. Vancomycin resistance in *S. aureus* has been previously reported in Tehran (Iran) by Emaneini *et al*. (23) whereas, published studies from other Iranian hospitals found vancomycin resistance as an extremely rare phenomenon (24, 25). Since no data is available on clinical usage of vancomycin and its efficacy, we cannot predict vancomycin resistance *in vivo*. Based on disk diffusion results, 55.5% of MRSA and 4.3% of MSSA strains were found intermediate resistant to vancomycin in our study. However, of these MRSA strains, five (6.2%) had MIC = 4 mg/L and the rest (29.6%) had MIC = 3 mg/L; thereby described as vancomycin-intermediate resistant *S. aureus* strains (VISA). Similarly 19 (23.4%) MSSA isolates were also observed to be VISA. Reduced susceptibility to vancomycin in *S. aureus* strains in our region is an alarm that may potentially drive the future development of vancomycin resistant strains.

In the present study, none of the MRSA isolates were found resistant to linezolid and teicoplanin, and all MSSA were susceptible to mupirocin, linezolid and teicoplanin. The complete susceptibility of MRSA and MSSA to linezolid and teicoplanin observed in this study is compatible with other published reports (26–28).

Three (4.34%) MSSA strain and four (4.93%) MRSA strains were found resistant to fusidic acid which is in accordance with studies reported from South Africa (19). It is well recognized that use of fusidic acid alone is associated with increased resistance as compared when added in combination with other drugs. Nathwani *et al*. (29) used fusidic acid in combination with rifampin and found more beneficial in treatment of serious MRSA infections. We observed rifampin resistance in 20.9% MRSA (14 strains with MIC = 32mg/L) and 5.79% MSSA isolates. This resistance rate was higher than that found in the studies reported by Askarian *et al*. (2). The higher rate of resistance to rifampin in our strains is probably due to the increasing usage of this antibiotic in our clinics for prophylactic and treatment purposes, especially for mycobacterial infections.

Mupirocin, an inhibitor of bacterial isoleucyl-tRNA synthetase, has potent activity against *S. aureus* strains including methicillin resistant *S. aureus* (MRSA) and glycopeptide intermediate *S. aureus* (GISA). It has been used to treat staphylococcal skin infections as well as to eliminate nasal carriage of MRSA. However, indiscriminate use of mupirocin has been reported to encourage the emergence of mupirocin-resistant *S. aureus* (30). Though mupirocin is used for a long time in our clinical set up, only one strain showed non susceptibility. The rate of mupirocin resistance in our study population corresponds more closely to a clinical report from a tertiary hospital in Pakistan (31). In spite of mupirocin resistance is not currently ascertained, still it is suggested that *S. aureus* isolates should be routinely tested in clinical microbiology laboratories in this region, so that mupirocin resistant isolates could be detected early, and to facilitate the prompt loss of the beneficial use of this antimicrobial agent against MRSA.

## CONCLUSION

In this study, linezolid and teicoplanin have shown to be the promising alternatives. Low level fusidic acid resistance was evident in our study. Mupirocin and fusidic acid are the cornerstones of MRSA eradication therapy and resistance to these antibiotics will affect the ability of hospitals to control the spread of MRSA. So their use should be restricted to where clinically indicated and where the infecting bacteria are susceptible. In addition, slight increase in vancomycin MIC towards intermediate level is an alarm which can be checked by repeated laboratory surveys and any elevated resistance should be managed in a best possible way.
